# A Question of Administration

**Published:** 1897-04-17

**Authors:** 


### A QUESTION OF ADMINISTRATION.

"Treasurer" writes: Will you permit me to ask through your columns for information on the following point in the case of a cottage hospital having seven beds? A committee, outside of the hospital committee, proposes to contribute the annual cost—say £80—of two beds, provided two specific beds are placed entirely at their disposal and only occupied by their nominees ; and this notwithstanding any demands which may be made on the accommodation of the institution from other sources. Is such a system in force anywhere, and how does it work ? If not in force, how do experienced managers think it would work ? I may add the hospital is not financially flourishing, and the proposed subsidy is a consideration.

